# Exceeding the Limits with Nutraceuticals: Looking Towards Parkinson’s Disease and Frailty

**DOI:** 10.3390/ijms26010122

**Published:** 2024-12-26

**Authors:** Martina Montanari, Nicola Biagio Mercuri, Giuseppina Martella

**Affiliations:** 1 Department of Systems Medicine, University of Rome Tor Vergata, Via Montpellier 1, 00133 Rome, Italy; m.montanari@hsantalucia.it; 2 Laboratory of Neurophysiology and Plasticity, IRCCS Fondazione Santa Lucia, 00179 Rome, Italy; 3 Neurology Unit, Policlinico Tor Vergata, University of Rome Tor Vergata, 00133 Rome, Italy; mercurin@uniroma2.it; 4 Department of Experimental Neuroscience, IRCCS Fondazione Santa Lucia, 00179 Rome, Italy; 5 Department of Wellbeing, Nutrition and Sport, Faculty of Humanities Educations and Sports, Pegaso Telematics University, 80145 Naples, Italy

**Keywords:** Parkinson’s disease, frailty, nutraceuticals, bioactive compounds, physiology and anatomy, antioxidants, inflammation, precision medicine

## Abstract

One of the most pressing challenges facing society today is the rising prevalence of physical and cognitive frailty. This geriatric condition makes older adults more vulnerable to disability, illness, and a heightened risk of mortality. In this scenario, Parkinson’s disease (PD) and geriatric frailty, which share several common characteristics, are becoming increasingly prevalent worldwide, underscoring the urgent need for innovative strategies. Nutraceuticals are naturally occurring bioactive compounds contained in foods, offering health benefits over and above essential nutrition. By examining the literature from the past decade, this review highlights how nutraceuticals can act as complementary therapies, addressing key processes, such as oxidative stress, inflammation, and neuroprotection. Notably, the antioxidant action of nutraceuticals appears particularly beneficial in regard to PD and geriatric frailty. For instance, antioxidant-rich nutraceuticals may mitigate the oxidative damage linked to levodopa therapy in PD, potentially reducing the side effects and enhancing treatment sustainability. Similarly, the antioxidant effects of nutraceuticals may amplify the benefits of physical activity, enhancing muscle function, cognitive health, and resilience, thereby reducing the risk of frailty. This review proposes a holistic approach integrating nutraceuticals with exercise, pharmacotherapy, and lifestyle adjustments. It promises to transform the management of ARD, prolong life, and improve the quality of life and well-being of older people.

## 1. Introduction

Over the past decade, the global population has experienced a notable increase in the average age, leading to a surge in the elderly demographic [1]. In 2020, the number of individuals aged 70 and older reached approximately 457.96 million worldwide (World Population Ageing 2020 Highlights). As people live longer, many face significant health challenges, with the majority suffering from one or more chronic, age-related diseases (ARDs), such as cardiovascular diseases, diabetes, neurodegenerative conditions, and frailty [2,3,4,5,6,7]. One major issue affecting this population is frailty, a clinically identifiable syndrome caused by the aging of multiple physiological systems, leading to increased vulnerability [8]. The development of frailty is multifactorial, with its underlying pathophysiology shaped by the interplay of various factors [8]. Frailty is commonly evaluated using two primary approaches: the frailty phenotype (FP) and the frailty index (FI) [9]. The FP identifies frailty in individuals who meet at least five criteria: weakness, slowness, low physical activity, self-reported exhaustion, and unintentional weight loss [10]. When only one or two of these criteria are present, the individual is classified as pre-frail, which indicates a higher likelihood of progressing to complete frailty [11]. On the other hand, the FI is a more comprehensive tool that assesses frailty through a 40-item evaluation, considering diseases, physical and cognitive impairments, psychosocial factors, and other geriatric syndromes [8]. Despite its thoroughness, the FI can be impractical for primary care practitioners to implement during routine bedside screenings [8]. A more feasible alternative is the Edmonton Frail Scale (EFS), which evaluates nine domains of frailty, including cognition, general health status, functional independence, social support, medication use, nutrition, mood, continence, and functional performance [12,13]. The EFS produces a score ranging from 0 to 17 and is a quicker and more accessible screening method [12,13]. Factors such as social isolation, depression, and a loss of independence also play a role in reducing both physical and mental activity, thereby worsening frailty [14]. Furthermore, difficulties in regard to eating and maintaining proper nutrition, often caused by motor or cognitive impairments, can result in malnutrition, which weakens physical health and facilitates the development of frailty [15]. However, frailty often emerges in neurodegenerative diseases as a result of the complex interactions among motor, cognitive, and physiological declines that characterize these conditions [8]. In disorders such as Parkinson’s disease (PD), motor dysfunction, manifested through symptoms like rigidity, slowed movements, and postural instability, significantly affects physical performance, leading to weakness and an elevated risk of falls, which are the main indicators of frailty [16]. Cognitive impairment in PD also plays a crucial role by reducing the ability to perform daily tasks, limiting self-care, and restricting participation in physical activities. These factors are vital in maintaining muscle strength and overall independence [17,18]. In addition, chronic inflammation, which is often associated with neurodegenerative diseases, accelerates muscle wasting and contributes to fatigue, further exacerbating frailty [19]. Not only is an inflammatory state commonly observed in both PD and geriatric frailty, but mitochondrial metabolic dysfunction also emerges as a shared characteristic. In PD, mitochondrial dysfunction leads to the loss of dopaminergic neurons, a defining feature of the condition [20]. Deficiencies in mitochondrial complex I, a key component of the electron transport chain, result in increased oxidative stress, mitochondrial fragmentation, and impaired metabolic function [21,22]. These changes accelerate neuronal degeneration and contribute to the motor symptoms in PD, such as tremors, rigidity, and postural instability [21,22]. In parallel, mitochondrial dysfunction is also a significant feature of geriatric frailty, which is linked to the natural age-related decline in mitochondrial efficiency [23]. This decline results in decreased energy production, increased oxidative stress, and impaired cellular function, contributing to muscle weakness, reduced endurance, and functional impairment, all characteristics of frailty [23]. Studies show that frail older individuals often exhibit lower mitochondrial content and diminished mitochondrial respiration in their skeletal muscles, negatively impacting muscle strength and overall physical performance [24,25]. The shared mitochondrial dysfunction seen in both PD and geriatric frailty suggests a common underlying mechanism that connects motor and cognitive impairment with physical decline [24,25]. These conditions frequently coexist, with frailty often exacerbating the symptoms and progression of PD, and vice versa [24,25]. This overlap highlights the potential for targeting mitochondrial health as a therapeutic strategy. Nutraceuticals, known for their antioxidant properties and ability to support cellular metabolism, offer a promising avenue for intervention [26]. By enhancing mitochondrial function, reducing oxidative stress, and supporting overall metabolic health, nutraceuticals could help slow the progression and alleviate the impact of PD and geriatric frailty, ultimately preserving physical and cognitive health in affected individuals [27]. Nutraceuticals, a term coined by Stephen DeFelice, are food-derived products offering health benefits beyond essential nutrition [1]. They encompass a wide range of products, including naturally nutrient-rich foods, like garlic, isolated nutrients, and herbal products [1]. Nutraceuticals, with their antioxidant, anti-inflammatory, and neuroprotective properties, offer promising adjuncts to conventional treatments [1]. In PD, compounds such as curcumin, resveratrol, and omega-3 fatty acids, have shown potential to slow disease progression and alleviate symptoms by targeting oxidative stress and mitochondrial dysfunction [1]. Similarly, in frailty, these substances may help enhance muscle function, reduce inflammation, and improve the overall resilience to physical and psychological stressors [1]. This review aims to explore the distinct effects of nutraceuticals on PD and frailty, evaluating their ability to influence the core mechanisms behind these conditions. Moreover, it underscores the importance of advancing the research and clinical approaches in this field, emphasizing the development of novel bioactive compounds, using cutting-edge delivery technologies, and incorporating personalized strategies based on genetic and epigenetic insights. These advancements will contribute to more precise, effective, and individualized treatments, allowing precision medicine to extend the health span [1].

## 2. Methods

To explore the rationale for linking PD and frailty, we aim to investigate the current research (approximately related to the last decade in terms of the signs of progress) indicating shared underlying mechanisms, outcomes, and possible connections related to interventions, such as nutraceuticals. The following are some factors that could provide a meaningful connection between PD and frailty:

- Shared pathophysiological mechanisms: inflammation and oxidative stress, mitochondrial dysfunction;
- Motor and functional decline: mobility and balance, sarcopenia, and muscle wasting;
- A risk of malnutrition;
- Cognitive decline and depression.

Developing a comprehensive and well-documented search formula is essential to ensure replicability and transparency in relation to the literature search.

PubMed serves as a critical source of biomedical research and will be used as the primary database for this search, given its extensive coverage of medical, biological, and life sciences literature. Target key terms and concepts for inclusion in the research questions: “Parkinson’s disease”, which encompasses synonyms and related terms such as “PD” or “parkinsonism” to capture all the relevant articles; “Frailty”, which is expanded with terms such as ‘frail elderly,’ ‘frailty syndrome,’ or ‘physical frailty’ to include variants used in the literature; “Nutraceuticals”, which includes such interrelated terms as “dietary supplements”, “functional foods”, or specific nutraceuticals (e.g., “omega-3”, “curcumin”) to cover various types of interventions. Our study focuses on recent advancements, limiting the search to studies published in English in the last 10 years.

We searched ClinicalTrials.gov https://clinicaltrials.gov/study/NCT06352099?cond=frailty&intr=Nutraceutical&rank=2 (accessed on 15 November 2024) to identify potential studies exploring the use of nutraceuticals in managing Parkinson’s disease and frailty.

## 3. Parkinson’s Disease vs. Frailty

PD and geriatric frailty are characterized by a gradual decline in physical and cognitive functions [28,29]. In both pathological conditions, the accumulation of senescent cells in tissues becomes more pronounced, leading to a heightened susceptibility to osteoarthritis, characterized by joint degeneration and impaired mobility [30,31]. PD and geriatric frailty also affect various organ systems, leading to physiological changes, such as reduced cell turnover, diminished function of mucous membranes, and muscle wasting. Furthermore, both pathological conditions lead to the gradual loss of muscle mass and strength (sarcopenia), impaired immune function, and increased vulnerability to infections and other illnesses [32,33,34]. Cognitive decline, ranging from mild memory lapses to severe forms of dementia, becomes more prevalent with age and is accompanied by reduced physiological resilience, making it harder for elderly individuals to recover from illness, injury, or stress [35,36,37]. PD and geriatric frailty diminish the quality of life of patients and increase the burden on the healthcare system, as elderly individuals often require more medical care and assistance [29]. Common conditions associated with aging include neurodegenerative diseases, such as PD and frailty [38]. These conditions diminish the quality of life of patients and increase the burden on the healthcare system, as elderly individuals often require more medical care and assistance [39].

### 3.1. Parkinson’s Disease

PD is a complex neurodegenerative disorder primarily characterized by the loss of dopaminergic neurons in the Substantia Nigra pars compacta (SNpc) and the accumulation of Lewy bodies (LBs) in the brain, which are aggregates of alpha-synuclein (αS) [40,41]. The diagnosis of PD is based on the patient’s history and a neurological examination [42,43]. Although primarily designed for research purposes, the diagnostic criteria established by the International Parkinson and Movement Disorder Society can aid clinicians in confirming the diagnosis [44,45]. The TRAP mnemonic can be helpful during diagnosis, as it includes tremors (T), rigidity (R), akinesia (A), and postural instability (P) [46,47]. However, PD also presents a wide range of less visible, non-motor symptoms, such as cognitive decline, depression, and pain, which contribute significantly to the overall disability experienced by patients [48,49]. These non-motor symptoms can be assessed using a specialized rating scale to quantify their burden on the patient [48,49]. Early indicators include symptoms like constipation (the most common early sign), acting out dreams during REM sleep (indicative of REM sleep behavior disorder), loss of smell (hyposmia), asymmetrical shoulder pain, and depression [50,51,52,53]. It is essential to recognize that general practitioners cannot be faulted for missing a diagnosis in the early stages, as these symptoms are often nonspecific and overlap with many other conditions [44,45]. The exact cause of PD remains elusive, but it is believed to result from a combination of genetic predispositions and environmental factors. Mutations in genes such as Leucine-rich repeat kinase 2 (LRRK2), Parkin7 (PARK7), and αS have been linked to familial forms of PD, while environmental exposure to toxins, such as pesticides, has been associated with an increased risk of the disease [54,55,56,57]. Additionally, mitochondrial dysfunction, oxidative stress, and neuroinflammation are critical contributors to the pathogenesis of PD. These factors lead to the accumulation of reactive oxygen species (ROS), which damage cellular components, further exacerbating neuronal death [58,59,60,61]. Dopaminergic agonists are a key therapeutic option in the treatment of PD [62,63]. Levodopa, the gold standard treatment for alleviating PD motor symptoms, is often administered with carbidopa, a peripheral dopa decarboxylase inhibitor, to increase levodopa’s availability in the brain, while reducing peripheral side effects, like nausea and vomiting [46,47].

Unlike levodopa, which needs to be converted into dopamine in the brain, dopamine agonists directly stimulate DA receptors, compensating for the dopamine deficit [62,63]. They are often used as an add-on with levodopa in advanced stages of the disease to manage motor fluctuations, such as “wearing-off” episodes [62,63]. While non-ergot DA agonists, including pramipexole, ropinirole, rotigotine, and apomorphine, are commonly used, their use increases ROS species [62,63]. Indeed, DA agonists are not without side effects; they can cause nausea, orthostatic hypotension, hallucinations, sleep disturbances, and impulse control disorders, such as compulsive gambling or hypersexuality, requiring careful monitoring [62,63]. Despite these risks, their ability to provide sustained symptom relief and improve motor function makes them essential in the comprehensive management of PD [62,63]. Dopaminergic agonists, while effective, are just one component of the broader therapeutic landscape for PD; understanding the current therapeutic approaches provides further insight into the various strategies used to manage the disease’s symptoms and progression [46,47,62,63].

#### 3.1.1. Current Therapeutic Approaches

Despite the wide range of treatment options, including pharmacological, non-pharmacological, and surgical interventions like brain, spinal, and vague nerve stimulators, patients still suffer from ongoing muscle weakness and no therapy has proven to be a definitive disease-modifying solution [62,63].

##### Conventional Pharmacological Treatments

Levodopa and Derivatives: Levodopa remains a central treatment for PD, being converted into dopamine in the brain to alleviate motor symptoms, like tremors and rigidity [64,65]. Its effectiveness diminishes over time, leading to side effects like dyskinesia [64,65]. Co-administration with carbidopa improves its efficacy and reduces peripheral side effects. However, as the disease progresses, the effectiveness of levodopa diminishes, and patients often experience motor fluctuations and dyskinesias (involuntary movements) [64,65,66].

MAO-B Inhibitors: Monoamine oxidase B inhibitors delay levodopa breakdown, extending its benefits in early-stage PD [67,68]. Although less potent than levodopa, they pose fewer risks of inducing dyskinesias [66]. These drugs are commonly combined with other therapies to enhance motor symptom management, especially as PD progresses [67,68,69].

COMT Inhibitors: Catechol-O-methyltransferase inhibitors increase levodopa’s availability in the brain by reducing its breakdown [69,70]. Drugs like entacapone and opicapone extend levodopa’s effects. However, they may cause adverse effects, such as dyskinesia and confusion. Tolcapone, though effective, is rarely used due to the risk of liver failure [69,70].

Anticholinergic Agents: These drugs, including trihexyphenidyl and benztropine, reduce tremors, particularly in younger patients [71,72]. However, their use is limited due to side effects like blurred vision and urinary retention [71,72].

##### Non-Conventional Pharmacological Treatments

Antidiabetic Agents: Medications such as glucagon-like peptide 1 (GLP-1) agonists and Dipeptidyl peptidase 4 (DPP-4) inhibitors may offer neuroprotective effects in PD by reducing neuroinflammation and oxidative stress [73,74,75]. Studies have shown potential benefits in regard to improving motor and cognitive symptoms in PD patients [76].

Intranasal Insulin: Insulin, administered intranasally, has shown promise in protecting dopaminergic neurons and improving motor function in PD patients, without affecting blood glucose levels [77,78].

Biguanides (Metformin): Although primarily used for treating type 2 diabetes, metformin has potential neuroprotective effects in PD [79,80]. Specific genetic polymorphisms in IL-1β and TNF-α genes may elevate PD risk [81], and metformin’s ability to reduce these pro-inflammatory cytokines could make it especially beneficial for PD patients with these specific gene variations [81]. Polymorphisms in Complex I, especially when coupled with pesticide exposure, are known to influence PD risk [81]. Since metformin acts as a Complex I inhibitor, it is plausible that particular Complex I gene variants could alter metformin’s impact on PD progression, particularly in individuals exposed to environmental toxins [81]. Moreover, metformin’s intracellular transport is mediated by organic cation transporters, which rely on their membrane concentrations to function effectively [81].

##### Non-Pharmacological Treatments

Stem Cell Therapy: Using pluripotent stem cells to regenerate damaged dopaminergic neurons is a promising future therapy for PD. Early trials using fetal cell transplants have shown long-term benefits, but also present risks like dyskinesia [82,83,84].

Gene Therapy: Gene therapies targeting defective genes like AADC and neurotrophic factors are being explored to modify disease progression in PD [85,86]. While promising in animal models, clinical application has faced gene distribution and efficacy challenges [85,86].

##### Surgical Treatments

Lesioning Procedures: Ablative surgeries, like pallidotomy and thalamotomy, target specific brain areas to alleviate motor symptoms [87]. Although effective, these procedures are reserved for patients that are unresponsive to medication, as they present the risk of neurological side effects [87].

Deep Brain Stimulation (DBS): DBS is widely used to control PD motor symptoms by delivering electrical impulses to the brain [88,89]. It improves motor function and reduces the reliance on medications, although it requires careful management to avoid side effects like dyskinesia and cognitive impairment [88,89].

Focused Ultrasound (FUS): FUS is a non-invasive method that uses ultrasound waves to target deep brain tissues, offering a promising alternative to traditional surgery for motor symptom relief in PD [90].

Gamma Knife Thalamotomy (GKT): GKT uses targeted gamma radiation to treat tremors in PD. It is minimally invasive, with fewer long-term complications, although risks such as radiation-induced neurological changes remain [91,92].

#### 3.1.2. Limitations of Conventional Treatments

Despite the availability of various treatments for PD, limitations and side effects persist across both pharmacological and non-pharmacological approaches [93]. Levodopa, a cornerstone therapy that is converted into dopamine to alleviate motor symptoms like tremors and rigidity, loses its effectiveness over time and can lead to side effects, such as dyskinesia and motor fluctuations [94]. While MAO-B inhibitors help delay levodopa’s breakdown and extend its benefits, they are less potent and are usually used in combination with other treatments [95]. COMT inhibitors increase levodopa availability, but may induce dyskinesia and confusion, with some drugs, like tolcapone, posing a risk of liver failure [96]. Anticholinergic agents, used for tremor control, particularly in younger patients, come with side effects like blurred vision and urinary retention, limiting their use [97]. Non-conventional pharmacological treatments also present challenges [93]. Antidiabetic agents, like GLP-1 agonists and DPP-4 inhibitors, have shown potential for reducing neuroinflammation, but their off-target effects remain under investigation [98]. Intranasal insulin may protect dopaminergic neurons and improve motor function, although it is still in the early-stage of research [98]. Metformin, an antidiabetic drug, has been associated with a reduced risk of PD, but raises concerns about vitamin B12 deficiency and possible cognitive decline. Non-pharmacological and surgical options carry their own risks [99,100]. Stem cell therapy is promising in regard to the regeneration of dopaminergic neurons, but carries the risk of dyskinesia and poses ethical challenges [101]. Gene therapy faces obstacles in regard to gene distribution and efficacy in clinical applications [102]. Surgical approaches, like lesioning procedures, are reserved for patients that are unresponsive to medication and come with neurological side effects [103]. DBS, although effective in controlling motor symptoms, can lead to dyskinesia and cognitive impairment [104]. Newer techniques, like FUS and GKT, offer less invasive alternatives, although they carry risks of tissue damage and radiation-induced neurological changes, respectively [105] (Table 1).

### 3.2. Frailty

Frailty is a clinical syndrome characterized by a reduction in physiological reserves and increased vulnerability to stressors, leading to adverse health outcomes, such as falls, hospitalization, disability, and death [107,108]. It is often seen in older adults and is associated with a decline in multiple body systems [107,108]. Clinically, frailty is diagnosed using criteria like the Fried frailty phenotype, which includes unintentional weight loss, self-reported exhaustion, weakness (grip strength), slow walking speed, and low physical activity [109,110]. The presence of three or more of these criteria indicates frailty, while one or two suggests a pre-frail state [111,112]. Frailty is not merely a consequence of aging, but rather a distinct clinical entity that significantly impacts an individual’s quality of life and functional independence [111,112]. It is associated with a higher risk of adverse outcomes, particularly in the presence of acute illnesses or surgical interventions [113]. Frailty is a growing global health issue, with significant consequences for healthcare systems and individual well-being. Defined by the deterioration of various physiological systems and a heightened susceptibility to external stressors, frailty dramatically increases the likelihood of mortality, hospitalization, and the need for long-term care, and reduces the patient’s quality of life. Research indicates a reciprocal association between severe depressive disorders and frailty, where each condition can exacerbate the risk of the other [114]. On one hand, frailty, a state of decreased physiological reserves and increased vulnerability to stressors, can heighten the risk of developing or experiencing worsening depressive symptoms due to its physical, social, and emotional burdens [114]. On the other hand, severe depressive disorders are linked to behaviors, hormonal imbalances, and inflammatory pathways that may accelerate frailty [114]. Antidepressant use, while essential in managing depressive symptoms, can also influence this relationship [115]. These medications may improve mental health and overall function, potentially mitigating frailty’s progression [115]. However, some antidepressants are associated with side effects, such as sedation, falls, or weight changes, which may inadvertently contribute to frailty in susceptible populations [115]. This interplay highlights the need for a personalized and cautious approach to managing depression in frail older adults, emphasizing both pharmacological and non-pharmacological interventions to optimize patient outcomes [115]. The pathophysiology of frailty is multifaceted, involving complex interactions between various biological systems [107,108]. Chronic inflammation plays a central role, with elevated levels of pro-inflammatory cytokines, such as inteleukin-6 (IL-6) and tumor necrosis factor-alpha (TNFα), contributing to muscle catabolism, reduced muscle mass, and strength, a condition known as sarcopenia, which is a core component of frailty [116,117,118]. Additionally, hormonal imbalances, including decreased levels of anabolic hormones, like testosterone and growth hormones, exacerbate the decline in muscle and bone health [119]. Mitochondrial dysfunction is also a critical factor in frailty, leading to decreased energy production and increased oxidative stress, which further accelerates cellular aging and tissue damage [120]. Additionally, frailty is associated with insulin resistance, dysregulated glucose metabolism, and impaired autophagy, all of which contribute to the decline in cellular and systemic resilience [121].

#### 3.2.1. Current Therapeutic Approaches

Patients affected by the clinical syndromes of frailty have limited options to effectively slow disease progression outside of exercise training [122]. Given the difficulty in reversing disability in older adults, its impact is both severe for individuals and costly for society [123]. Therefore, developing new strategies to maintain functional capacity and independence in later life is crucial, particularly in the context of chronic illness [123]. A combined approach involving exercise, nutrition, and pharmacological interventions may help mitigate the onset and progression of frailty [124].

##### Drug Therapy

Hormone Therapies: Among the potential pharmacological treatments extensively studied in preclinical settings are hormone therapies and myostatin inhibitors [125]. Hormone therapies include the administration of testosterone, growth hormones (GHs), ghrelin, insulin, and thyroid hormones [126,127,128,129]. Testosterone replacement therapy, given its known metabolic and anabolic effects, has been explored as a possible treatment for frailty [130]. Clinical trials have shown that testosterone can modestly improve muscle function and overall physical capacity in frail patients [131,132]. However, side effects like the risk of prostatic hyperplasia necessitate further large-scale studies to validate its safety and efficacy [133]. While showing promise in preclinical studies for its anabolic, anti-inflammatory, and antioxidant benefits, GH therapy has yet to demonstrate clinical effectiveness [125]. Ghrelin is another potential treatment, due to its ability to stimulate the patient’s appetite and enhance gastric motility [134].

Insulin: By increasing amino acid delivery and intramuscular blood flow, insulin promotes muscle protein synthesis [135]. The thyroid hormone, a critical metabolic regulator targeting skeletal muscle, has been linked to muscle wasting and diminished function in overt and latent thyroid dysfunction cases [136].

Myostatin: Myostatin, a cytokine within the transforming growth factor-β (TGF-β) family, is highly expressed in skeletal muscles and regulates muscle growth [137,138]. However, several trials involving myostatin inhibitors have yielded underwhelming results, showing limited therapeutic benefit [139]. Nevertheless, a study involving bimagrumab, a myostatin inhibitor, demonstrated positive outcomes, improving both functional capacity and independence in elderly sarcopenic individuals [140].

GDF-15: Another promising target has recently gained attention, growth differentiation factor 15 (GDF-15), a key regulator in muscle pathophysiology and a global stress mediator [141,142]. Evidence indicates that GDF-15 is associated with reduced muscle mass, impaired performance, and heightened inflammation [143]. Neutralizing GDF-15 has shown promise in reversing these effects, helping to restore muscle function and physical capacity [144]. In experimental models, anti-GDF-15 treatment significantly increased muscle mass by boosting the patient’s appetite and food intake, leading to improved physical function [145].

Exercise: Exercise is now recognized as the most effective therapy for slowing the progression of frailty [146,147]. Well-structured and closely supervised exercise training programs are designed to combat muscle atrophy, stimulate muscle growth, and preserve muscle function, as individuals age [146,147]. Resistance exercise benefits muscle health through various physiological mechanisms and signaling pathways, including vasodilation, antithrombotic effects, reduced oxidative stress, anti-inflammatory responses, activation of the mechanistic target of rapamycin complex-1 (mTORC1), enhanced mitochondrial biogenesis, increased Insulin-like Growth Factor 1 (IGF-1), stimulation of peroxisomes, and improved insulin sensitivity [148,149]. These molecular adaptations show that the skeletal muscle is highly responsive and adaptable to activity. Low-intensity training enhances mitochondrial efficiency and oxygen utilization, while high-intensity exercise stimulates muscle cell proliferation and increases contractile protein production [150]. Exercise also upregulates gene transcription related to calcium (Ca2+) signaling via the Adenosine monophosphate-activated protein kinase (AMPK) pathway, influencing the energy status of muscle cells [151].

Nutrition: Malnutrition encompasses various forms of undernutrition, such as wasting, stunting, the patient being underweight, vitamin and mineral deficiencies, obesity, and related non-communicable diseases [152]. Nutritional deficiencies in terms of micronutrients (e.g., vitamins and minerals) and macronutrients (such as energy stores and substrates) contribute to a worsening catabolic state in conditions like frailty [153,154,155]. For example, vitamin D deficiency can impair muscle function, alter calcium flow, and promote inflammation. However, it also reduces muscle mass and contributes to poor physical performance in older adults [156]. Consequently, vitamin D supplementation is potentially a therapeutic option for managing frailty [157,158]. New insights into the link between muscle health and nutrition reveal that proper nutrition supports muscle function, stimulates muscle growth (anabolism), and regulates muscle protein synthesis, glucose, insulin levels, and neuromuscular and vascular functions [159]. Nutrition also plays a crucial role in nutrient sensing, mitochondrial efficiency, and communication between muscles and the immune system. When combined, nutritional interventions and exercise can have additive effects, particularly when resistance training is paired with protein supplementation, improving muscle mass and function [160]. Dietary protein is vital for maintaining muscle structure, function, and a healthy balance between anabolic and catabolic processes in frail elderly individuals [161]. Essential amino acids, like leucine, trigger strong anabolic responses by activating muscle signaling pathways that enhance mRNA translation and muscle protein synthesis [162]. Omega-3 polyunsaturated fatty acids also offer a potential benefit, due to their anti-inflammatory properties [163]. Despite their crucial role in managing frailty, nutritional interventions face challenges. These include the complexity of food and nutrient interactions, difficulty in blinding treatments, low patient adherence, and the influence of confounding factors like ethnicity, genetics, and physiological condition, along with dietary habits and food culture variations [152].

#### 3.2.2. Limitations of Conventional Treatments

Pharmacological and nutritional interventions for treating frailty come with notable limitations and potential side effects [164]. Testosterone therapy, despite its ability to improve muscle function, carries risks, such as prostatic hyperplasia, necessitating large-scale trials to confirm its safety [165]. Though promising in preclinical studies, GH treatment has yet to demonstrate clinical efficacy [126,127,128,129]. Ghrelin and insulin therapies, while showing potential in regard to improving muscle function and protein synthesis, may pose risks, with insulin linked to poorer outcomes in heart failure (HF) patients with diabetes [134]. Thyroid hormone interventions face challenges in managing muscle wasting, particularly in cases of thyroid dysfunction [165]. Myostatin inhibition trials, including those involving bimagrumab, have yielded limited therapeutic benefits and further research is needed [166]. Nutritional interventions, like protein and vitamin D supplementation, also require careful consideration, particularly in frail patients with chronic kidney disease or heart failure [167]. Additionally, compliance with dietary interventions remains low, and factors like ethnicity, genetics, and food culture introduce significant variability, making treatment outcomes less predictable [168] (Table 2).

## 4. Unveiling the Differences: Nutraceuticals vs. Conventional Food

Interest in nutraceuticals and functional foods is increasing, driven by ongoing research into their properties and applications and increasing consumer demand [169,170]. “Functional foods” and “nutraceuticals” are often used interchangeably, contributing to confusion, due to their overlapping characteristics [169,170]. Functional foods are broadly defined as “foods and food components that provide health benefits beyond basic nutrition”, as stated by the Institute of Food Technologists (IFT) [171]. These foods are typically consumed as part of a regular diet and include naturally occurring bioactive substances, such as dietary fiber or enriched components, like probiotics and antioxidants [169,170]. In contrast, nutraceuticals are formulated products that provide health benefits through specific doses and formats, such as capsules, tablets, or powders, often more closely resembling pharmaceutical products [172]. While functional foods and nutraceuticals offer health-promoting effects, their distinction lies in their format and regulatory classification. Functional foods are consumed as ordinary foods and often lack specific dosing requirements [173,174]. Conversely, nutraceuticals are designed to address specific health conditions or preventive needs with precise compositions, making them distinct from conventional dietary sources [173,174]. Globally, regulatory frameworks highlight these differences. For instance, in Japan, functional foods are categorized under the Food for Specified Health Use (FOSHU) program, which emphasizes scientifically validated health benefits [175,176,177]. In the U.S., the FDA regulates functional foods and nutraceuticals under the Federal Food, Drug, and Cosmetic Act and the Dietary Supplement Health and Education Act (DSHEA). However, they are not classified as medicines [178,179]. Similarly, in Europe, functional foods are defined by their ability to improve health and reduce disease risk, as part of the General Food Law and Framework Directive [180,181,182,183]. By consolidating these definitions, we emphasize that functional foods are primarily dietary components with health benefits when consumed regularly, while nutraceuticals are more targeted, formulated interventions. Clear distinctions are essential to avoid confusion in research, regulatory contexts, and consumer education [184,185].

### 4.1. Categories of Major Nutraceuticals

The growing interest in nutraceuticals stems from their potential to improve individuals’ quality of life, addressing modern challenges and consumer demand for alternative forms of healthcare [172]. Regardless of their origin, these compounds can provide various health benefits, from antioxidant and anti-inflammatory properties, to supporting specific health conditions [186]. Despite their potential, nutraceuticals face several challenges. Their diverse composition and varied modes of action make it difficult to develop standardized delivery methods [187]. Additionally, their low bioavailability and potential interactions with other food components hinder their practical use [187]. To address these challenges, researchers are exploring encapsulation technologies [187]. By encapsulating nutraceuticals within protective structures, it is possible to improve their stability, solubility, and bioavailability [184,185]. Various methods, including micro and nano-encapsulation, are being investigated to optimize the delivery of these valuable compounds [184,185]. Ultimately, the successful development and utilization of nutraceuticals depend on a clear understanding of their properties, effective delivery systems, and robust regulatory frameworks [187]. Nutraceuticals are categorized according to their applications into various classes, including traditional, non-traditional, fortified, recombinant, phytochemicals, herbal products, functional foods, dietary supplements, probiotics, and prebiotics [188]. Each class of nutraceuticals relates to distinct applications and offers distinct benefits, depending on its specific characteristics [188]. The categories include conventional foods, fortified, enriched, enhanced, and dietary supplements [188].

#### 4.1.1. Traditional Nutraceuticals

##### Functional Foods

Functional foods contain ingredients that enhance antioxidant and anti-inflammatory activities [182,189]. Examples of functional foods include rice, wheat, beans, soybeans, lentils, chocolate, citrus fruits, nuts, and fermented milk [182,189]. Rice, for instance, is a staple food rich in carbohydrates and low in fat, salt, and sugar. It also contains resistant starch, which supports gut health [190]. Similarly, wheat is valued for its fiber-rich bran, which promotes gastrointestinal health [191]. Other examples of such foods, like carrots and broccoli, contain active components such as sulforaphane and lycopene, which are known for their health benefits [192]. However, more scientific studies are needed to validate the health claims on these product labels, including in regard to the following aspects:

- Carotenoids: Carotenoids are natural pigments found in plants, fruits, vegetables, and algae, known for their antioxidant and anti-inflammatory properties [193]. These compounds, including β-carotene and lutein, offer various health benefits, such as improving vision, cognitive function, and heart health, while helping prevent cancer [194]. Their antioxidant activity is due to their chemical structure, which allows them to neutralize free radicals [182,189,193,194];
- Collagen hydrolysate: Collagen hydrolysate, derived from collagen found in animal connective tissues, has several health benefits, including antioxidant, anti-aging, and anti-inflammatory effects [195,196]. Studies have shown that collagen hydrolysate can boost the immune system, improve skin hydration elasticity, and reduce wrinkles, especially in cases of photoaged skin [197,198];
- Dietary fibers: Dietary fibers are non-digestible carbohydrates found in vegetables, fruits, and whole grains [191]. They are classified into soluble and insoluble fibers, each offering specific health benefits [199]. For example, soluble fibers can help manage digestive health by delaying gastric emptying, while insoluble fibers can alleviate constipation [191]. High-fiber diets are also linked to a reduced risk of inflammatory bowel diseases [191];
- Fatty acids: Fatty acids in oils, fats, and fish supplements are crucial for energy storage and offer anti-inflammatory and immune-boosting benefits [200]. Omega-3 polyunsaturated fatty acids (PUFAs), in particular, have been shown to reduce the severity of symptoms in conditions like rheumatoid arthritis, when taken in sufficient doses [201];
- Phytochemicals: Phytochemicals are bioactive compounds derived from plants that support various biochemical and metabolic functions in the body [202]. They offer neuroprotective benefits and can reduce the risk of cancer, heart disease, and neurodegenerative disorders through their antioxidant properties [202];
- Herbs: Herbs like garlic, ginger, and aloe have been used for centuries for their health benefits, which include reducing cholesterol, promoting wound healing, and offering antioxidant properties [203]. The effectiveness of herbs can vary depending on how they are processed and consumed [203];
- Probiotics: According to the World Health Organization, probiotics are defined as “live microorganisms which, when administered in adequate amounts, confer a health benefit on the host” https://ehpm.org/wp-content/uploads/2024/07/EHPM_Probiotics_Guidelines_2022_digital_v02.pdf (accessed on 15 November 2024). They are commonly found in fermented foods, especially dairy products, that promote digestive health and support the immune system [204]. Lactobacillus, Bifidobacterium, and Streptococcus are among the most commonly used probiotic strains, known to maintain a healthy balance of gut bacteria [204];
- Prebiotics: Prebiotics are non-digestible ingredients that stimulate the activity of probiotics in the gut [205]. They act as a fertilizer for beneficial gut bacteria, enhancing the health benefits provided by probiotics [206]. Fructo-oligosaccharides and inulin are prebiotics used in functional foods to improve digestive health [207];
- Dietary supplements: Dietary supplements, available in various forms like tablets, capsules, and powders, are intended to supplement the diet and ensure adequate nutrient intake [208]. Joint supplements include omega-3 fatty acids, vitamins, and minerals, which can prevent nutrient deficiencies and support overall health [209,210].

#### 4.1.2. Non-Traditional Nutraceuticals

Non-traditional nutraceuticals are artificially synthesized food products that enhance health through biotechnology and agricultural breeding [188]. These nutraceuticals can be categorized into fortified and recombinant types, based on how they are processed [188]. Examples include rice enriched with β-carotene and cereals fortified with vitamins and minerals, which boost antioxidant activity and provide essential nutrients, like provitamin A [188].

- Fortified nutraceuticals: Fortified nutraceuticals are foods enhanced with additional vitamins or micronutrients to improve their nutritional value [211]. For instance, orange juice fortified with calcium or milk enriched with vitamin D, which helps prevent deficiencies and supports overall health [212]. Such products can also offer specific benefits, like enhanced glycemic control when calcium is added to orange juice [213].
- Recombinant nutraceuticals: Recombinant nutraceuticals are genetically modified foods created through biotechnology to include beneficial compounds [214,215]. Examples include iron-fortified rice, golden rice, and multivitamin corn [216]. These products contain genes that enhance their nutritional content, such as increasing the levels of vitamins, carotenoids, and proteins [217,218]. Gold kiwifruit, for example, has been modified to boost its vitamin C, carotenoid, and lutein content, making it a rich source of essential nutrients [219].

## 5. Mechanisms of Nutraceutical Action in Geriatric Frailty and Parkinson’s Disease

Nutraceuticals are believed to enhance human health, extend life expectancy, and delay the onset of aging and chronic diseases [220]. Numerous nutraceutical supplements have demonstrated positive effects on conditions like PD and frailty [8,221]. Their ability to address mitochondrial dysfunction, first, and also oxidative stress, inflammation, mitochondrial dysfunction, and protein aggregation, underscores their potential as complementary strategies for promoting cellular health and healthy aging and mitigating disease progression [222]. The primary challenge in managing both PD and frailty lies in addressing their clinical symptoms, while minimizing the side effects of pharmacological treatments, particularly concerning mitochondrial bioenergetics. Patients with these conditions often require multiple medications, which can negatively affect mitochondrial function by promoting oxidative stress and inflammation.

In PD, mitochondrial dysfunction is a well-documented issue and treatments like the use of dopaminergic agents (e.g., levodopa) can further damage mitochondria by increasing oxidative stress and mitochondrial fragmentation [223,224]. In the context of geriatric frailty, polypharmacy is also common, and the concurrent use of multiple medications often exacerbates mitochondrial impairment, resulting in higher levels of oxidative stress and inflammation. These effects contribute to the progression of frailty, weakening muscle function and the patient’s overall resilience [225]. The influence of pharmacological treatments on mitochondrial health highlights the critical need for a more strategic approach to medication management [223,224]. Clinicians should carefully evaluate pharmacological interventions, seeking to optimize treatments, while considering alternatives that protect mitochondrial function, reduce oxidative damage, and minimize inflammation, ensuring better long-term outcomes for patients with PD and geriatric frailty [223,224]. Nutraceuticals are believed to enhance human health, extend life expectancy, and delay the onset of aging and chronic diseases [220]. Numerous nutraceutical supplements have had positive effects on conditions like PD and frailty [8,221]. Their ability to address oxidative stress, inflammation, mitochondrial dysfunction, and protein aggregation underscores their potential as complementary strategies for promoting healthy aging and mitigating disease progression [222].

### 5.1. Anti-Inflammatory Activity

Nutraceuticals are known for their anti-inflammatory properties, which are crucial for preventing and treating diseases associated with chronic inflammation [226]. One significant advantage of using nutraceuticals as anti-inflammatory agents is that they can complement traditional anti-inflammatory drugs, enabling lower drug dosages to be used and reducing potential side effects [227].

Chronic inflammation is a leading cause of several major diseases, including frailty and PD [228,229]. Nutraceuticals can help mitigate this type of inflammation by suppressing inflammatory cytokines, like interleukins, tumor necrosis factor-alpha (TNF-α), and cyclooxygenase-2 (COX-2) [230]. For example, curcumin, the active compound in turmeric, has potent anti-inflammatory properties. It works by inhibiting key inflammatory pathways, including nuclear factor kappa B (NF-κB) and COX-2 pathways, and reducing the production of pro-inflammatory cytokines, like TNF-α, interleukins-6 (IL-6), and IL-1β. These cytokines are implicated in muscle degradation and systemic inflammation in frailty [231,232]. Despite its apparent pharmacokinetic limitations, curcumin, a well-known anti-inflammatory compound, has been shown to exhibit a wide range of pharmacological activities and has demonstrated effectiveness against numerous diseases [233]. These include its anticarcinogenic effects [234], hepatoprotective properties [235], thrombosuppressive action [236], cardioprotective benefits [237], antiarthritic effects [238], and its role in combating infections [239]. The study of the chemical biology of aging is expected to reveal candidate compounds and fundamental mechanisms that will drive the development of treatments for age-related diseases [240]. Curcumin exemplifies this concept due to its multiple in vitro benefits. It has been shown to extend the lifespan in *C. elegans* and *Drosophila*, although similar effects have not been observed in mice [241,242]. Still, considerable evidence suggests that curcumin may aid in treating neurodegenerative and other age-related diseases, potentially enhancing the health span [243]. Polyunsaturated fatty acids (PUFAs) are another class of nutraceuticals that effectively manage inflammatory disorders. Docosahexaenoic acid (DHA) and eicosapentaenoic acid (EPA) are known to reduce inflammation by inhibiting the production of pro-inflammatory cytokines and eicosanoids, such as prostaglandins and leukotrienes [244]. They also promote the production of specialized pro-resolving mediators (SPMs), like resolvins and protectins, which help combat inflammation [245]. PUFA treatment has been shown to decrease the expression of NF-κB and reduce pro-inflammatory markers, while increasing anti-inflammatory markers like IL-10 in patients with conditions such as Duchenne muscular dystrophy [246]. Additionally, DHA has demonstrated neuroprotective effects in various animal models of neurodegenerative diseases [247,248]. While there is less research on DHA consumption and its impact on PD, recent epidemiological studies suggest that a high intake of unsaturated fatty acids may lower the risk of developing PD and offer protection against pesticide-induced neurotoxicity [247,248]. Research involving the MPTP animal model of PD has also highlighted the protective effects of PUFAs against MPTP-induced neurotoxicity [249]. Although the exact mechanisms behind these effects are not fully understood, several studies have shown that PUFAs enhance the release of neurotrophic factors, regulate genes involved in oxidative stress and apoptosis, and reduce inflammation associated with PD [250]. Polyphenols are bioactive compounds found in fruits, vegetables, and teas [251,252]. They exhibit strong anti-inflammatory and antioxidant activities by modulating signaling pathways, like NF-κB and Nrf2, and reducing oxidative stress [251,252]. Resveratrol, found in red grapes, and quercetin, found in apples and onions, specifically inhibit inflammatory mediators and support muscle health [251,253]. Indeed, it activates SIRT1 and improves mitochondrial function, protecting against cognitive decline [254]. Resveratrol, found in red grapes, has shown promise in terms of improving mitochondrial function and reducing oxidative stress, both critical in regard to muscle preservation and physical resilience associated with frailty [251,253]. Abundant in onions, apples, and tea, quercetin combats inflammation and enhances muscle strength. Studies suggest it can ameliorate sarcopenia, a key component of frailty. Found in green tea, catechins exhibit anti-inflammatory properties and support vascular function, contributing to overall physical robustness in humans. In dark-colored berries (e.g., blueberries, blackberries), anthocyanins promote vascular health and cognitive function. Derived from turmeric, curcumin reduces inflammation and oxidative stress, improving muscle and joint health. This is particularly beneficial in preventing frailty-related mobility limitations. Found in pomegranates and nuts, these compounds foster gut health by enhancing microbiota composition and reducing systemic inflammation. Lycopene (LYC), a natural carotenoid pigment primarily found in red fruits and vegetables, such as tomatoes, papayas, pink grapefruits, pink guavas, and watermelons, has garnered significant attention for its diverse biological activities [255,256]. LYC is an unsaturated acyclic carotenoid, with eleven linear conjugated and two non-conjugated double bonds [257]. Studies have demonstrated that LYC exhibits potent antioxidant and anti-inflammatory properties, both in vitro and in vivo, and it can also cross the blood–brain barrier [258,259]. Furthermore, higher serum levels of carotenoid pigments, like lycopene, lutein, and zeaxanthin, have been associated with a reduced risk of neurodegenerative diseases [260]. The discovery of a pro-inflammatory shift in the gut microbiota associated with PD and its potential involvement in the progression of this neurodegenerative disorder has sparked interest in exploring gut microbiota-modulating treatments, such as probiotics, as possible therapeutic options for PD [41,261]. Probiotics provide these health benefits through various mechanisms, such as restoring the balance to a disrupted intestinal microbiome [262], enhancing the function of the intestinal barrier [263], and activating enzymes that produce metabolites, which help to regulate both peripheral and central energy metabolism and inflammation, in addition to promoting neurogenesis, neurotransmission, and even behavioral changes [264]. Animal studies of PD, for instance, have demonstrated that probiotics can lower the level of inflammatory cytokines, like IL-1β and IL-6, which in turn helps prevent neuroinflammation [265]. Indeed, probiotics exhibit anti-inflammatory effects by modulating the NF-κB signaling pathway, inflammatory cytokines, and the regulatory T-cell response [266]. A combination of probiotics, such as *Lactobacillus rhamnosus*, *Bifidobacterium lactis*, and *Bifidobacterium longum,* has been shown to induce IL-10 production and reduce pro-inflammatory cytokines [267,268]. Prebiotics, like β-(1,3)-glucan, also demonstrate anti-inflammatory and immunomodulatory effects [269]. In animal studies, pre-treatment with β-(1,3)-glucan prevented symptoms of inflammatory bowel disease and inhibited inflammatory cytokines and reactive oxygen species (ROS) [270]. Other nutraceuticals, including ginger, cinnamon, and peppermint, also possess potent anti-inflammatory activities [271]. Emerging evidence from both in vivo and in vitro studies highlights the neuroprotective properties of ginger and its vital active components, namely zingerone, 6-shogaol, and 6-gingerol, in regard to PD [272]. These protective effects are primarily linked to the regulation of neuroinflammation, oxidative stress, intestinal permeability, dopamine synaptic transmission, and potentially mitochondrial dysfunction [272]. Several transcription factors and signaling pathways are involved in mediating these benefits, including NF-κB, p38 mitogen-activated protein kinase (MAPK), phosphatidylinositol-3-kinase (PI3K)/Akt, extracellular signal-regulated kinase (ERK) 1/2, and AMP-activated protein kinase (AMPK)/proliferator-activated receptor gamma coactivator one alpha (PGC1α) [273]. These pathways contribute to ginger’s neuroprotective effects in PD [272]. Similarly, cinnamon and peppermint extracts have shown strong anti-inflammatory effects, by significantly reducing the expression of inflammatory cytokines IL-1 and IL-6, respectively, in experimental animal models and individuals with various CNS complications, like PD and frailty [274]. Ginkgolides, bioactive compounds derived from the Ginkgo biloba tree, have been used in traditional Chinese medicine for centuries [275]. Extensive research has validated their neuroprotective properties, making them a valuable component of treatments for various neurological disorders, including PD [275]. Ginkgolides exert a multifaceted influence on the CNS. They modulate neurotransmitter activity, such as glutamate and dopamine, and inhibit platelet-activating factors (PAFs), a critical inflammatory mediator [275]. These actions contribute to their neuroprotective effects [275].

### 5.2. Antioxidant Activity

Curcumin increases antioxidant defense mechanisms by upregulating transcription and expression levels of antioxidant enzymes and improving mitochondrial function [276]. In vitro studies have shown that curcumin presents senolytic properties, causing a reduction in some hallmarks of senescence (i.e., p16, IL-6, IL-8, MMP3, and MMP13) [277]. However, curcumin has low bioavailability, which compromises its senolytic activity [278]. In combination with piperine, alginates, or nanocapsules, the stability and bioavailability of curcumin is improved [279]. PD is characterized by a chronic, low-grade inflammatory process, in which activated microglia release cytotoxic compounds, most notably peroxynitrite, that contribute to the death and dysfunction of nearby dopaminergic neurons [280]. As neurons die, they release damage-associated molecular pattern proteins, like the high mobility group of proteins, which further activates microglia through various receptors, amplifying the inflammatory response [281]. Since peroxynitrite is central to this destructive cycle, nutraceutical approaches that either reduce microglial peroxynitrite production or enhance the scavenging of peroxynitrite-derived oxidants could be valuable for preventing and managing PD [281]. Peroxynitrite formation can be mitigated by inhibiting microglial NADPH oxidase activity, which produces its precursor, superoxide, or by downregulating signaling pathways that stimulate the microglial expression of inducible nitric oxide synthase (iNOS) [281]. Nutrients and compounds, such as phycocyanobilin from spirulina, ferulic acid, long-chain omega-3 fatty acids, adequate vitamin D levels, hydrogen sulfide-promoting substances like taurine and N-acetylcysteine, caffeine, epigallocatechin-gallate, butyrogenic fiber, and probiotics, may help reduce microglial iNOS induction [282]. Additionally, scavenging peroxynitrite-derived radicals can be enhanced through supplementation with zinc or inosine. Astaxanthin may protect the mitochondrial respiratory chain from peroxynitrite damage and environmental toxins [280]. Plant-based diets low in protein and possibly diets rich in corn and spermidine might offer protection by enhancing mitophagy and supporting mitochondrial health. Furthermore, low-protein diets can help maintain a more stable response to levodopa therapy [280]. Exogenous antioxidants, like vitamin C, E, and phenolic compounds, are crucial in neutralizing free radicals [283]. In contrast to traditional antioxidants like vitamins C, E, and β-carotene, natural compounds such as flavonoids (quercetin, curcumin, luteolin, and catechins) and magnolol/honokiol have demonstrated superior efficacy in inhibiting oxidative processes in various in vitro and in vivo models of aging and PD [283]. Vitamin C is highly effective at scavenging harmful free radicals, such as hydroxyl and superoxide anion radicals, and helps protect cells and DNA from oxidative damage [284]. Alongside vitamin C, vitamin E also contributes to safeguarding cells by preventing lipid peroxidation [285]. Gingerols, the bioactive compounds found in ginger, have demonstrated various neuroprotective properties, including antioxidant and anti-amyloidogenic properties [286]. Moreover, 6-Gingerol, a key component of ginger, has been shown to inhibit astrocyte overactivation and reduce inflammation in microglia [287]. Both environmental and genetic factors, including iron accumulation and oxidative stress, contribute to PD development [55]. Through its active compounds, ginger may offer potential benefits for individuals with PD [272]. Ginger could potentially mitigate cognitive dysfunction associated with this condition by inhibiting inflammation, increasing the nerve growth factor, and promoting synapse formation [272]. In conclusion, the antioxidant properties of nuts offer a promising approach to mitigating the health challenges associated with aging, making them a valuable dietary addition for older individuals [288].

Saffron, a prized spice derived from the Crocus sativus plant, has long been valued for its culinary and cosmetic applications [289]. Recent research has unveiled its potential therapeutic benefits, particularly in regard to neurological disorders [289]. Saffron’s antioxidant properties have shown promise in mitigating the effects of neurodegenerative conditions [289]. Saffron and its components have been found to enhance antioxidant defenses against reactive oxygen species, lipid peroxidation, and other oxidative damage [289]. While preclinical studies have provided encouraging results, further clinical research is essential to fully elucidate the mechanisms underlying saffron’s antioxidant actions and to validate its potential as a therapeutic agent for neurological disorders [289].

### 5.3. Promoting Healthy Aging

PD and geriatric frailty are often viewed as age-related conditions (ARDs), and it is essential to acknowledge the potential role of nutraceuticals in mitigating the effects of aging [290]. Research indicates that specific nutraceuticals, particularly those with antioxidant and anti-inflammatory properties, can help address the cellular and molecular damage associated with the aging process [290]. As a result, these compounds may contribute to reducing the severity of neurodegenerative diseases, such as PD, and geriatric frailty [8]. Nutraceuticals have the potential to support mitochondrial function, decrease oxidative stress, and enhance cellular resilience, which are crucial factors in maintaining overall health in aging individuals [291]. By targeting these cellular mechanisms, nutraceutical interventions may offer a promising approach to improving physical function, cognitive health, and overall well-being in populations affected by PD and geriatric frailty, ultimately promoting healthier and more active aging [221]. Emerging evidence supports the ability of different phytochemical classes to modulate the senescence process, underscoring the importance of nutraceutical research for promoting healthy aging [292]. Data on the anti-aging effects of various natural and synthetic compounds are available from databases like Geroprotectors (http://geroprotectors.org/resources (accessed on)) and DrugAge (https://ngdc.cncb.ac.cn/databasecommons/database/id/4466 (accessed on. The scientific evaluation of the anti-aging effects of natural compounds is still in the early stages, and evidence regarding their senolytic properties is limited [293]. Tocotrienols, a member of the vitamin E family, possess antioxidant properties and play a role in cell signaling, immune responses, and apoptosis [294]. Recently, they have gained attention for their senolytic properties, stimulating senescence in cancer cells and reducing the accumulation of senescent cells in healthy tissues, thereby slowing the aging process [295]. Combining quercetin and dasatinib has significantly enhanced the health span in various mouse models [296]. Derived from Piper longum, Piperlongumine (PL) is known for its anticancer properties. It suppresses cancer stemness and has been shown to preferentially kill senescent human fibroblasts, making it a promising anticancer agent, with potential senolytic effects [293].

## 6. Emerging Nutraceuticals and Future Directions

The field of nutraceuticals is rapidly evolving, with novel compounds and advanced technologies paving the way for more effective anti-aging interventions [187,297]. Novel compounds with potential anti-aging effects are at the forefront of current research [293]. For instance, pterostilbene, a compound structurally similar to resveratrol, but with superior bioavailability, is gaining attention for its potent antioxidant and anti-inflammatory properties, which could play a crucial role in slowing the aging process and combating neurodegenerative diseases [298]. Similarly, urolithin A, a metabolite derived from ellagitannins that is found in pomegranates, has shown promise in enhancing mitochondrial function and promoting mitophagy, thereby supporting cellular health and longevity [299]. Overall, the integration of nanotechnology into nutraceutical formulations is overcoming the barriers to implementation [300]. Nanoparticles, liposomes, and nanoemulsions are employed to encapsulate bioactive compounds, protecting them from degradation and improving their absorption and bioavailability [300,301]. For example, nanocurcumin, a nanoparticle form of curcumin, has enhanced stability and bioavailability, leading to more pronounced anti-inflammatory and neuroprotective effects [241]. These cutting-edge delivery systems could revolutionize the effectiveness of nutraceutical interventions, making them more potent and reliable for preventing and managing age-related diseases [241,278]. Moreover, the future of nutraceuticals is moving towards personalized interventions, tailored to an individual’s genetic and epigenetic profile [302]. As our understanding of genomics and epigenetics deepens, it is becoming increasingly clear that the efficacy of nutraceuticals can vary significantly based on an individual’s unique genetic makeup [302]. For instance, specific gene variants may influence how well a person metabolizes specific nutrients, impacting the effectiveness of nutraceuticals like omega-3 fatty acids or polyphenols [303]. By integrating genetic testing and epigenetic analysis, healthcare providers could tailor nutraceutical regimens to optimize their anti-aging effects [304]. This personalized approach could also involve monitoring epigenetic markers, such as DNA methylation patterns or microRNA expression, to adjust nutraceutical interventions dynamically, ensuring they remain effective as an individual ages [304]. In summary, the future of nutraceuticals lies in developing novel bioactive compounds, applying advanced delivery technologies, and shifting toward personalized interventions based on genetic and epigenetic data [305]. These advancements promise to significantly enhance the role of nutraceuticals in promoting healthy aging and preventing age-related diseases, offering a more precise, effective, and individualized approach to health span extension [187,297]. Indeed, a recent study, currently in the recruitment phase and listed on ClinicalTrials.gov https://clinicaltrials.gov/study/NCT06352099?cond=frailty&intr=Nutraceutical&rank=2 (accessed on 15 November 2024) led by Dr. Giovannini Silvia, aims to investigate the effects of Altemor^®^, a supplement with diosmin, hesperidin, and herbal extracts, on cognitive function, balance, fatigue, and quality of life in older adults. Aging involves gradual physical and cognitive changes driven by cellular and molecular shifts that reduce functional ability, contributing to frailty, falls, and disability. Nutrition plays a crucial role in counteracting frailty. Diets rich in plant-based foods provide macronutrients, micronutrients, and phytochemicals, including beneficial compounds like phenols and flavonoids. These phytochemicals support health through their antioxidant, anti-inflammatory, and cardiovascular benefits and may protect against age-related cognitive decline. Flavonoids, such as those in rutin, hesperidin, and diosmin, are particularly active, with some already used in supplements for cardiovascular and cognitive health. This focus on Altemor^®^ is significant as it builds on existing research on the individual components of the supplement and it is the first study to investigate the combined effects of these components. The study will explicitly assess Altemor^®^’s potential to support blood microcirculation and address age-related challenges.

## 7. Challenges and Limitations vs. Advantages and Benefits

### 7.1. Challenges and Limitations

While nutraceuticals hold great promise for promoting health and combating age-related diseases, several challenges and limitations must be addressed to realize their full potential [305,306]. A primary concern is the bioavailability and pharmacokinetics of nutraceuticals [307]. Many bioactive compounds in nutraceuticals, such as polyphenols, curcumin, and omega-3 fatty acids, have inherently low bioavailability due to poor absorption, rapid metabolism, and quick elimination from the body [307]. For example, despite its potent anti-inflammatory and antioxidant properties, curcumin is notorious for its poor bioavailability, as it is quickly metabolized in the liver and intestines [308]. Nutraceuticals typically have lower and more variable bioavailability compared to pharmaceutical drugs, due to factors like poor absorption, rapid metabolism, and limited cellular uptake. Studies have highlighted that bioactive compounds in nutraceuticals, like polyphenols and carotenoids, often face difficulties in relation to being absorbed efficiently through the gastrointestinal tract, with a significant portion being metabolized before reaching systemic circulation [308]. In contrast, pharmaceutical drugs are meticulously designed to optimize bioavailability through advanced delivery systems, such as nanoparticles or liposomes, which enhance absorption and facilitate targeted cellular uptake [308]. These systems ensure that drugs are delivered efficiently to specific sites of action, thus improving therapeutic efficacy. However, the bioavailability of nutraceuticals within cells is often hindered by their molecular characteristics, such as poor solubility and instability, which necessitate the development of novel formulations, including nanocarriers, to improve their cellular delivery and effectiveness [309]. Consequently, nutraceuticals tend to exhibit a slower onset and less precise targeting than pharmaceutical drugs, limiting their immediate therapeutic impact [309]. This limitation severely reduces its effectiveness when consumed orally, leading to the need for higher doses or the development of advanced delivery systems, such as nanoparticles or liposomes, to enhance absorption and prolong circulation in the bloodstream [308]. Additionally, the pharmacokinetics of nutraceuticals, which involve their absorption, distribution, metabolism, and excretion, can vary widely among individuals due to their age, genetics, gut microbiota composition, and overall health [310]. This variability complicates the standardization of dosing regimens and makes it challenging to predict therapeutic outcomes consistently [310]. Another significant issue is the safety and long-term efficacy of nutraceuticals [310]. Although generally regarded as safe due to their natural origin, the long-term use of specific nutraceuticals may carry risks, particularly at high doses or in combination with other medications [310]. For instance, prolonged high-dose consumption of certain antioxidants, like vitamin E, has been associated with an increased risk of hemorrhagic stroke, highlighting the need for caution and proper dosage guidelines [311]. Moreover, the long-term efficacy of nutraceuticals remains an open question. While short-term studies often demonstrate beneficial effects, robustness in regard to clinical trials still needs to be improved to confirm that these benefits persist over years or decades of use [310]. The potential for cumulative side effects or interactions with other dietary supplements or medications over prolonged periods must be explored [310]. This knowledge gap underscores the necessity for more extensive longitudinal studies to assess both the safety and sustained effectiveness of nutraceuticals in diverse populations [310]. Lastly, the regulatory and ethical considerations surrounding nutraceuticals present significant challenges [183]. The regulatory landscape for nutraceuticals varies considerably between countries, with some regions having stringent regulations similar to those for pharmaceuticals, while others offer minimal oversight [183]. Nutraceuticals are often classified as dietary supplements rather than drugs, meaning they are not subject to the same rigorous testing of their efficacy, safety, and quality [183]. This can lead to inconsistencies in product quality, including variations in the concentration of active ingredients or the presence of contaminants. Furthermore, the marketing of nutraceuticals often includes claims that are not fully supported by scientific evidence, potentially misleading consumers about their health benefits [183]. Ethical concerns also arise from the commercialization of nutraceuticals, mainly when vulnerable populations are targeted with exaggerated promises of anti-aging or disease-preventive effects [183]. As the industry grows, there is a pressing need for more stringent regulations to ensure product safety, efficacy, and accurate labeling, as well as for ethical guidelines to govern the marketing and distribution of these products [183].

While nutraceuticals hold great promise for promoting health and combating age-related diseases, several challenges and limitations must be addressed to realize their full potential [305,306]. A primary concern is the bioavailability and pharmacokinetics of nutraceuticals [307]. Many bioactive compounds in nutraceuticals, such as polyphenols, curcumin, and omega-3 fatty acids, have inherently low bioavailability due to poor absorption, rapid metabolism, and quick bodily elimination [307]. For example, despite its potent anti-inflammatory and antioxidant properties, curcumin is notorious for its poor bioavailability, as it is quickly metabolized in the liver and intestines [308]. This limitation severely reduces its effectiveness when consumed orally, leading to the need for higher doses or the development of advanced delivery systems, such as nanoparticles or liposomes, to enhance absorption and prolong circulation in the bloodstream [308].

Additionally, the pharmacokinetics of nutraceuticals, which involve their absorption, distribution, metabolism, and excretion, can vary widely among individuals due to age, genetics, gut microbiota composition, and overall health [310]. This variability complicates the standardization of dosing regimens and makes it challenging to predict therapeutic outcomes consistently [310]. Another significant issue is nutraceuticals’ safety and long-term efficacy [310]. Although generally considered safe due to their natural origin, the long-term use of specific nutraceuticals may carry risks, particularly at high doses or in combination with other medications [310]. For instance, prolonged high-dose consumption of certain antioxidants like vitamin E has been associated with an increased risk of hemorrhagic stroke, highlighting the need for caution and proper dosage guidelines [311]. Moreover, the long-term efficacy of nutraceuticals remains an open question. While short-term studies often demonstrate beneficial effects, the robustness of clinical trials still needs to be improved to confirm that these benefits persist over years or decades of use [310]. The potential for cumulative side effects or interactions with other dietary supplements or medications over prolonged periods must be explored [310]. This gap in knowledge underscores the necessity for more extensive longitudinal studies to assess both the safety and sustained effectiveness of nutraceuticals in diverse populations [310]. Lastly, nutraceuticals’ regulatory and ethical considerations present significant challenges [183]. The regulatory landscape for nutraceuticals varies considerably between countries, with some regions having stringent regulations similar to those for pharmaceuticals while others offer minimal oversight [183]. Nutraceuticals are often classified as dietary supplements rather than drugs, meaning they are not subject to the same rigorous testing for efficacy, safety, and quality [183]. This can lead to consistency in product quality, with variations in the concentration of active ingredients or the presence of contaminants. 

Furthermore, the marketing of nutraceuticals often includes claims not fully supported by scientific evidence, potentially misleading consumers about their health benefits [183]. Ethical concerns also arise from the commercialization of nutraceuticals, mainly when vulnerable populations are targeted with exaggerated promises of anti-aging or disease-preventive effects [183]. As the industry grows, there is a pressing need for more stringent regulations to ensure product safety, efficacy, and accurate labeling, as well as for ethical guidelines to govern the marketing and distribution of these products [183].

### 7.2. Advantages and Benefits

Nutraceuticals have gained in popularity due to their perceived safety, widespread availability, and ability to enhance immune functions effectively. These bioactive compounds, when consumed in controlled doses, offer synergistic health benefits, without significant toxicity, as long as proper guidelines are followed [187]. Despite their cost, nutraceuticals are gaining recognition as valuable therapeutic agents, due to their potent antioxidant and anti-inflammatory properties. These compounds can counteract the harmful effects of oxidative stress and chronic inflammation, often exacerbated by conventional drug therapies [215]. By mitigating oxidative damage, nutraceuticals can enhance the efficacy of standard treatments and support improved long-term outcomes [215]. In the management of PD, the integration of nutraceuticals with treatments such as levodopa has shown promise [215]. Nutraceuticals may extend the duration of the drug’s effectiveness, often referred to as the “dopamine honeymoon” phase, while reducing the risk of developing secondary dyskinesias [312]. Similarly, in the context of frailty, nutraceuticals have been found to limit mitochondrial damage caused by polypharmacy, a common issue in the treatment of comorbidities [312]. By protecting mitochondrial health, these compounds help preserve physical functions and slow the progression of functional decline [313]. The growing body of evidence underscores the role of nutraceuticals not only as adjuncts to existing therapies, but also as tools to address the underlying mechanisms of oxidative stress, inflammation, and mitochondrial dysfunction, offering a multifaceted approach to managing PD and geriatric frailty [226]. Despite their cost, nutraceuticals are establishing themselves as valuable therapeutic agents due to their potent antioxidant and anti-inflammatory properties. These compounds can reverse the damaging effects of oxidative stress and chronic inflammation, often exacerbated by conventional drug therapies [170,313,314,315]. By alleviating oxidative damage, nutraceuticals can enhance the efficacy of regular treatments and promote improved long-term outcomes [170,313,314,315]. Supplementing nutraceuticals with treatments such as levodopa has shown promise in managing PD. Nutraceuticals can extend the duration of drug efficacy, often called the “dopamine honeymoon phase,” while reducing the risk of developing secondary dyskinesias [170,313,314,315]. Similarly, in the context of frailty, nutraceuticals have been seen to limit mitochondrial damage caused by polypharmacy, a common problem in treating comorbidities. By protecting mitochondrial health, these compounds help preserve physical function and slow the progression of functional decline [170,313,314,315]. The growing body of evidence underscores the role of nutraceuticals not only as adjuncts to existing therapies but also as tools to address the mechanisms underlying oxidative stress, inflammation, and mitochondrial dysfunction, offering a multifaceted approach. According to this we can propose a new type of classification of nutriacetucals based on their application (Table S1 supplementary data) [316,317,318,319,320]. Advanced delivery technologies, such as nanoparticles and liposomes, further enhance their bioavailability and effectiveness, paving the way for tailored therapeutic strategies [170,313,314,315]. 

## 8. Conclusions

PD and geriatric frailty are distinct pathologies, yet they exhibit significant overlaps, particularly within the context of advancing age. While frailty predominantly affects older adults, PD typically manifests in later life, often in the final decades. With the increase in life expectancy globally, the emphasis has shifted from merely prolonging life to enhancing its quality for individuals living with these conditions. Although halting the neurodegenerative processes or reversing neuronal loss in PD remains unattainable, nutraceuticals have emerged as a promising adjunctive strategy. These compounds, known for their antioxidant and anti-inflammatory properties, have the potential to mitigate the cellular damage driving symptom progression in both PD and geriatric frailty, offering improved outcomes and a better quality of life for patients [321]. In addition to reducing oxidative stress and inflammation, nutraceuticals can target mitochondrial dysfunction, a critical factor in the pathophysiology of both conditions. By preserving mitochondrial health, they may slow cognitive and physical decline, enhancing the patient’s resilience against disease progression [322]. This review highlights the role of nutraceuticals as a potential complement to conventional therapies. When combined with pharmacological treatments, physical exercise, and lifestyle modifications, nutraceuticals can help modulate antioxidant, inflammatory, and neuroprotective pathways, potentially enhancing the effectiveness of traditional management strategies. In PD, specific nutraceuticals can address oxidative stress and support neuroprotection, thereby improving clinical outcomes. Similarly, in frailty, nutraceuticals can help counteract muscle weakness, cognitive impairment, and overall functional decline. Integrating nutraceuticals into a comprehensive treatment strategy that includes exercise and targeted drug therapy may not only improve functional health, but also extend the lifespan and enhance well-being in aging populations. However, several challenges remain regarding their use, including the need to optimize their bioavailability, ensure their long-term safety, address the related regulatory frameworks, and refine formulations for maximum accuracy and efficacy. Future research should focus on determining optimal dosages, evaluating the synergistic effects of nutraceuticals with conventional therapies, and understanding their role within holistic care approaches. In summary, combining nutraceuticals with physical activity, pharmacological interventions, and lifestyle changes has the potential to transform the management of ARDs, fostering not only longer lives, but also healthier, more fulfilling ones for older individuals.

## Figures and Tables

**Table 1:** Overview of therapeutic approaches to PD.

Category	Treatment	Mechanism	Benefits	Limitations/Side Effects	Ref.
	**Levodopa and** **Derivatives**	Converted into dopamine to alleviate motor symptoms.	Effective for tremors and rigidity.	Diminished effectiveness over time, dyskinesia, motor fluctuations.	[64,65,66]
**Conventional Pharmacological Treatments**	**MAO-B Inhibitors**	Delays breakdown of levodopa, extending benefits.	Fewer patients develop dyskinesias, used in early-stage PD.	Less potent than levodopa, often used in combination with other therapies.	[64,65,66,67,68,69]
	**COMT Inhibitors**	Increases levodopa availability by reducing breakdown.	Extends levodopa’s effects.	Dyskinesia, confusion, tolcapone risk of liver failure.	[96]
	**Antidiabetic Agents**	May reduce neuroinflammation and oxidative stress.	Potential neuroprotective effects, motor and cognitive improvements	Potential off-target effects; still under study.	[73,74,75,76]
	**Biguanides (Metformin)**	Potential neuroprotective effects.	Neuroprotective effects in PD.	Risk of vitamin B12 deficiency, potential cognitive decline.	[79,80,106]
**Non-Pharmacological Treatments**	**Stem Cell Therapy**	Regenerates dopaminergic neurons.	Long-term motor benefits.	Risk of dyskinesia, ethical concerns, early-stage research.	[82,83,84]
	**Gene Therapy**	Targets defective genes and neurotrophic factors.	Promising disease-modifying potential.	Gene distribution challenges, efficacy concerns in regard to clinical application.	[85,86]
	**Lesioning Procedures**	Targets specific brain areas to alleviate motor symptoms.	Effective for motor symptom relief.	Neurological side effects, reserved for medication-unresponsive patients.	[87]
**Surgical Treatments**	**DBS**	Delivers electrical impulses to control motor symptoms.	Improves motor function, reduces medication reliance.	Risk of dyskinesia, cognitive impairment, requires careful management.	[88,89]
	**FUS**	Non-invasive ultrasound used to target brain tissue.	Promising alternative to traditional surgery.	Still under study, potential for tissue damage.	[90]
	**GKT**	Uses gamma radiation to treat tremors.	Minimally invasive, fewer long-term complications.	Radiation-induced neurological changes possible.	[91,92]

**Table 2:** Overview of therapeutic approaches to frailty.

Category	Treatment	Mechanism	Benefits	Limitations/Side Effects	Ref.
**Hormone Therapy**	**Testosterone**	Increases anabolic and metabolic activity, promoting muscle growth and improving physical capacity.	Modestly improves muscle function and overall physical capacity in frail patients.	Requires large-scale studies for safety and efficacy validation. Risk of prostatic hyperplasia.	[67,68,69,131,132,133]
	**GH**	Anabolic, anti-inflammatory, and antioxidant effects in preclinical models.	Shows promise in preclinical studies in regard to muscle growth and function.	Has not demonstrated clinical effectiveness. Uncertain due to lack of clinical efficacy data.	[125]
	**Ghrelin**	Stimulates appetite and enhances gastric motility.	Potential to improve muscle mass and nutritional status by stimulating appetite.	Clinical benefits are not fully validated.	[134]
	**Insulin**	Promotes muscle protein synthesis by increasing amino acid delivery and blood flow to muscles.	Enhances muscle protein synthesis and may prevent muscle wasting.	Associated with poorer outcomes in heart failure patients with diabetes. Risk of adverse effects in patients with heart failure.	[73,74,75,135,136]
	**Thyroid Hormones**	Critical metabolic regulator affecting skeletal muscle.	Linked to improved muscle metabolism and function.	Limited effectiveness in cases of overt and latent thyroid dysfunction. Potential to worsen muscle wasting in thyroid dysfunction cases.	[64,65,66,136]
	**Myostatin**	Blocks myostatin, a cytokine that regulates muscle growth, to promote muscle mass increase.	Positive outcomes in improving muscle function and independence in elderly sarcopenic individuals.	Limited therapeutic benefits found in many clinical trials. Unknown practical value due to limited clinical success.	[137,138]
	**GDF-15**	Neutralizes GDF-15, which is associated with reduced muscle mass and heightened inflammation, to restore muscle function.	Significantly increases muscle mass, boosts appetite, and improves physical function in experimental models.	Experimental models developed so far; requires further validation in clinical settings.	[137,138,145]
**Exercise**	**Resistance Training**	Involves mTORC1 activation, mitochondrial biogenesis, increased IGF-1, and enhanced insulin sensitivity, reducing oxidative stress and inflammation.	Preserves and enhances muscle mass, strength, and function in frail individuals.	Requires structured programs and close supervision, making adherence challenging. Risk of injury in frail patients if not supervised properly.	[146,147,148,149]
**Nutrition**	**Vitamin D**	Regulates calcium flow and reduces inflammation, impacting muscle function.	May improve muscle mass and physical performance in older adults with a deficiency.	Effectiveness limited by patient adherence and variability in dietary habits. Uncertain in patients with chronic kidney disease or heart failure.	[156]
	**Protein**	Stimulates muscle protein synthesis and anabolism, particularly through essential amino acids like leucine, and inflammation.	Helps maintain muscle structure and function, and improves muscle mass in frail elderly individuals	Compliance issues due to variability in diet, ethnicity, and genetics.	[146,147,160,161]
	**Omega-3 Fatty Acids**	Anti-inflammatory properties that support muscle health.	May reduce inflammation and support muscle function in frailty.	Challenges include low adherence and complex interactions with other nutrients.	[146,147,152,163]

## Data Availability

All the data shown in this paper are available in the PubMed Library. The authors created all the representative drawings appositely and they are available on request.

## Supplementary Materials

The following supporting information can be downloaded at: https://www.mdpi.com/article/10.3390/ijms26010122/s1.
