# Supplementary material for: Exceeding the Limits with Nutraceuticals: Looking Towards Parkinson’s Disease and Frailty
Source: Int J Mol Sci. 2024 Dec 26;26(1):122. doi: 10.3390/ijms26010122 (PMC11719863; doi:10.3390/ijms26010122)
Supplement: Supplementary file 1 [file ijms-26-00122-s001.zip › ijms-3347007-supplementary.pdf]

Table S1. Proposed classification of nutraceutical substances on the bases of their function.

Classification Criteria	Subcategories	References
**Bioactive Components**	Phenolic compounds, Alkaloids, Terpenoids, Peptides	[316]
**Functional Activities**	Antioxidant, Anti-inflammatory, Anti-cancer, Antimicrobial	[317]
**Source-based Classification**	Plant-based, Animal-based, Marine-based, Microbial-based	[318]
**Delivery Methods and Composition**	Oral, Topical, Sublingual, Injectable, Nanoparticles	[319]
**Health Benefits and Disease Prevention**	Cardiovascular, Cognitive, Bone Health, Diabetes, Skin	[320]
